# Clinical Relevance of Minimal Residual Viremia during Long-Term Therapy with Nucleos(t)ide Analogues in Patients with Chronic Hepatitis B

**DOI:** 10.1371/journal.pone.0067481

**Published:** 2013-06-27

**Authors:** Melanie Maier, Uwe G. Liebert, Christian Wittekind, Thorsten Kaiser, Thomas Berg, Johannes Wiegand

**Affiliations:** 1 Universitätsklinikum Leipzig, Institut für Virologie, Leipzig, Germany; 2 Universitätsklinikum Leipzig, Institut für Pathologie, Leipzig, Germany; 3 Universitätsklinikum Leipzig, Institut für Laboratoriumsmedizin, Klinische Chemie und Molekulare Diagnostik, Leipzig, Germany; 4 Universitätsklinikum Leipzig, Department für Innere Medizin, Dermatologie und Neurologie, Klinik und Poliklinik für Gastroenterologie und Rheumatologie, Leipzig, Germany; Saint Louis University, United States of America

## Abstract

**Background:**

Successful therapy of chronic hepatitis B with nucleos(t)ide analogues (NUCs) has been defined by undetectable HBV-DNA determined with conventional PCR (lower limit of detection (LLD) 60–80 IU/mL) in clinical registration trials. However, current EASL guidelines recommend highly sensitive real-time PCR (LLD<10–20 IU/mL) and define treatment response by HBV-DNA<10 IU/mL.

**Aim:**

We evaluated frequency and relevance of minimal residual viremia (MRV) during long-term NUC-treatment in a real-life setting.

**Methods:**

Frozen serum samples (HBV-DNA negative by in-house PCR, LLD <73 IU/mL) were re-analyzed by real-time PCR (LLD<10 IU/mL, Abbott, Germany). MRV was defined by real time PCR positivity and conventional PCR negativity.

**Results:**

237 samples of six HBsAg carriers and 27 NUC-treated CHB patients were analyzed (treatment period 28 (11–111) months, different treatment regimens with mono- or combination therapy). MRV was detected in 31/33 individuals (n = 160/237 serum samples) and more frequent in HBsAg carriers (95%) and HBeAg positive (87%) compared to HBeAg negative patients (53%) (p<0.0001, respectively). Five HBsAg carriers, five HBeAg positive, and four HBeAg negative individuals were continuously HBV-DNA positive. MRV was not significantly more often observed during NUC-monotherapies compared to combination therapies. Concomitant immunosuppressive therapy was present in nine cases and did not influence the results. Viral resistance occurred in three immunocompetent patients with adefovir or lamivudine monotherapy.

**Conclusions:**

MRV is frequently observed during long-term NUC-therapy. Adjustment of treatment with highly potent NUCs does not seem to be necessary in case of minimal residual viremia in a real-life setting.

## Introduction

Suppression of viral replication below the threshold of HBV-DNA assays is defined as treatment success of nucleos(t)ide analogues (NUCs) in patients with chronic hepatitis B virus (HBV) infection. The optimal treatment duration has not been discovered yet, because viral rebound is commonly observed after cessation of NUCs [1], [2]. Initially, conventional polymerase chain reactions (PCR) with a lower limit of detection (LLD) between 60–80 IU/mL were applied to define treatment success [3]–[6]. During long-term therapy with entecavir and tenofovir more than 90% of patients achieve viral suppression below the PCR threshold defined in the registration trials [7]–[9]. Subsequent studies have proven the potency of both drugs to suppress viral replication even below the lower limit of detection of modern highly sensitive PCRs (12–19 IU/mL) [8], [10]. Successful HBV-DNA suppression detected with conventional as well as with real-time PCR leads to histological improvement of HBV induced liver disease [8], [10], [11]. However, a clear cut-off of HBV-DNA reduction which is associated with histological improvement is not well defined, although disease progression below an HBV-DNA level of 2000 IU/mL is unlikely [12]. In contrast, in case of ongoing viral replication development of viral resistance against nucleos(t)ide analogues is foreseeable, especially during monotherapy with drugs with low barrier to resistance [13].

Current European treatment guidelines recommend treatment monitoring with highly sensitive real-time instead of previously available conventional PCRs and define long-term treatment response by HBV-DNA<10–20 IU/mL. In case of partial virologic response [detectable HBV-DNA by real-time PCR during continuous NUC therapy) and adequate patients compliance it is recommended to either switch to a more potent drug or to add a more potent substance without cross-resistance [2]. However, recent publications have challenged these switch- or add-on strategies: Patients with only partial virologic response after 48 weeks of treatment reach HBV-DNA levels<80 IU/mL during prolonged entecavir monotherapy and do not develop entecavir resistance [14]. A combination therapy with tenofovir plus emtricitabine is not more effective in adefovir treated patients than tenofovir alone [15], and even a combination of entecavir plus tenofovir does not achieve a statistically higher suppression of HBV-DNA<50 IU/mL than a monotherapy with entecavir in treatment-naïve individuals [16]. Thus, it seems questionable whether a switch to a more potent drug or an add-on strategy is always necessary or whether minimal residual hepatitis B viremia can be tolerated in real-life without compromising efficacy and safety of continuous NUC therapy.

Cut-off values for the definition of treatment response may not be ideally defined, because nucleos(t)ide analogue treated patients usually remain HBsAg positive and therefore detection of HBV-DNA may only be dependent on the sensitivity of the applied assays. After invention of highly sensitive PCRs observation of viral blips may not be uncommon, and it should be determined which level of minimal viral replication is associated with viral resistance. We therefore evaluated frequency and clinical relevance of minimal residual viremia (real time PCR positive, conventional PCR negative) during long-term treatment with nucleos(t)ide analogues in a real life setting of a tertiary referral centre.

## Methods

### Ethical Statement

The patients provided written informed consent prior to the experiments of the study. The protocol and the informed consent procedure were approved by the ethics committee of the University of Leipzig (ethical vote number 246-12-02072012).

### Patients and Methods

Prospectively collected stored frozen serum samples of HBV mono-infected patients without concomitant liver diseases treated with long-term NUC therapy (HBV-DNA negative by in-house PCR, LLD<73 IU/mL) were re-analyzed with a real-time PCR (LLD<10 IU/mL, Abbott, Germany). None of the analysed serum samples was collected during an interferon alfa based treatment regimen.

For the Abbott real-time PCR, serum samples were automatically extracted by magnetic particles on the m2000sp system with an input volume of 500 µL and an elution volume of 70 µL. 50 µL were further used for PCR on the m2000rt system. For sample results less than LLD (<10 IU/mL), but positive, IU values were calculated from sample Ct using test specific calibration curve equation. Retrospective analysis with the highly sensitive method also revealed samples with values above the lower-detection limit of the in-house-test. This is in concordance with previous findings, that in real-life settings, low input volumes in extraction as well as PCR lead to a higher variation and often lower test sensitivity [17]. The robustness of highly sensitive real-time tests with high input volumes favours these tests for reliable therapy monitoring.

Minimal residual viremia was defined as a real time PCR positive, but in-house PCR negative HBV-DNA result. A virological breakthrough was considered if HBV-DNA levels increased>1 log_10_ to a HBV-DNA level>100 IU/mL determined by real-time PCR in two consecutive serum samples and showed a continuous increase.

In-house quantification of HBV DNA and simultaneous screening for YMDD-motif mutations were done using a real-time protocol and FRET (fluorescence resonance energy transfer) hybridization probes (LightCycler 1.2 or 2.0 instrument, Roche Molecular Systems, Mannheim, Germany). Consensus primers (Metabion, Martinsried, Germany) HBV1 (5′-AAATTCGCAGTCCCMAAYC-3′) and HBV2 (5′GACAAAAGAAAATTGGTAAMAGYGG-3′) were used to amplify a fragment of approximately 512 bp of the RT/HBsAg. A set of 4 fluorescence-labeled hybridization probes (TibMolbiol, Berlin, Germany) was designed to allow quantification (HBV1Q 5′-GGATGTGTCTGCGGCGTTTTATCAT-FL-3′ and HBV2Q 5′-LCR640-GGATGTGTCTGCGGCGTTTTATCAT-ph-3′) with monitoring of fluorescence signal during annealing phase of each amplification cycle (channel setting 640 nm/530 nm) as well as mutational analysis (HBV YMDD1 5′- AGGGCTTTCCCCCACTGTTTGGCTTTCAG-FL-3′ and HBV YMDD2 5′-LCR705-TATATGGATGATGTGGTATTG-ph-3′) by melting curve analysis (channel setting 705 nm/530 nm). 200 µL of serum was automatically extracted using magnetic beads (MagnaPure LC System, Roche Molecular Systems, Mannheim, Germany) and eluted in a volume of 100 µL. 5 µL of DNA were further used for amplification. PCR reaction was done in 20 µL glass capillaries containing master mix at a final concentration of 4 mM MgCl_2_, 1-fold butter, 100 ng/µL BSA (Sigma, St. Louis, USA), 10 pmol of each primer (metabion, Martinsried, Germany), 3 pmol of each hybridization probe, 250 µM dNTP’s (Roche, Molecular Systems, Mannheim, Germany), and 2 U Platinum TaqPolymerase (Invitrogen, Karlsruhe, Germany). The cycle conditions were as follows: initial denaturation and enzyme activation at 95°C for 40 sec, 45 cycles of denaturation at 95°C for 0 sec, primer annealing at 58°C for 10 sec with single fluorescence detection and extension at 72°C for 15 sec. Melting curve analysis consisted of product denaturation at 95°C for 30 sec, cooling to 35°C for 30 sec with (20°C/sec), and ramping to 85°C at a temperature transition rate of 0.1°C/sec with continuous monitoring of fluorescence. Viral load was determined by external standard curve analysis with a standard range of 75-10^10^ IU/mL and a linear detection range of 300 to 10^9^ IU/mL. For mutation analysis of rt AS204, hybridization probe HBVYMDD2 perfectly matches wild-type sequence M204M (ATG) resulting in a melting peak of 59°C, whereas M204V (GTG) results in a shift to 55°C, YIDD (ATT) to 52°C. Samples with an additional mutation at rt 207 already show lower melting points in wild type and are not suitable for the sequence approach.

For HBV genotyping and further mutational analysis direct sequencing was applied. Primer HBV1 and HBV3 (5′-GCAGCAAAGCCCAAAAGACC-3′, 1022-1003) were used to amplify a fragment of approximately 716 bp. The patient derived sequence of 677 bp corresponds to RT codon 67–291 and HBsAg codon 58–226. In short, PCR was run on a block-cycler system in a volume of 50 µL with a final concentration of 1.5 mM MgCl, 1-fold butter, 10 pmol of each primer (Metabion, Martinsried, Germany), 250 µM dNTP’s (Roche, Molecular Systems, Mannheim, Germany), and 2 U Platinum TaqPolymerase (Invitrogen, Karlsruhe, Germany) and 5 µL template DNA. Initial denaturation at 95°C for 2 min was followed by 45 cycles of denaturation at 95°C for 30 sec, annealing at 58°C for 30 sec and elongation at 72°C for 2 min with a final elongation at 72°C for 9 min. In case of viral load below detection limit of in-house real-time PCR of 73 IU/mL, a semi-nested protocol was used. A first-round PCR-step was added with primers HBV0 (5′ CTCGTGGTGGACTTCTCTC-3′) and HBV3 with the same PCR conditions. 1 µL of first-round PCR were subjected to the above described protocol. After gel purification of the product with the Wizard SV Gel and PCR Clean-Up System (Promega, Mannheim, Germany), PCR Primers and the DyeTerminator v1.1 Cycle Sequencing Kit (PE Applied Biosystems, Foster City, CA, USA) were used to sequence the fragment in both directions on an ABI Prism 310 Genetic Analyzer (PE Applied Biosystems, Foster City, CA, USA). HBV-genotypes were determined by geno2pheno (http://hbv.bioinf.mpi-inf.mpg.de/, MPI Saarbrücken, Germany) and resistance and immune escape were analyzed by the HBV tool of HIV-GRADE (http://www.hiv-grade.de, Germany).

The HBeAg status was determined by a chemiluminescence magnetic microparticle-based immunoassay (CMIA) (ARCHITECT® HBeAg Assay, Abbott Diagnostics, IL, USA) according to the manufacturer’s instructions (samples with S/CO values greater or equal 1.0 were considered to be reactive).

The clinical course of patients was analysed by laboratory assessments (alanine-aminotransferase (ALT), bilirubin, prothrombin time) and ultrasound evaluation. Disease deterioration was defined as increase of ALT values>2x baseline values, increase of bilirubin levels above the upper limit of normal, or decrease of prothrombin time below 70% (lower limit of normal).

Data analysis was performed using Excel (Microsoft, Redmond, WA, USA). Statistical differences were analysed by Fisheŕs exact test (GraphPad Software, La Jolla, CA, USA). A p-value<0.05 was considered as statistically significant.

## Results

### Prevalence of Minimal Residual Viremia

237 serum samples of 27 patients with chronic HBV infection treated with nucleos(t)ide analogues and six untreated inactive HBsAg carriers were analysed. Baseline characteristics and different long-term sequential treatment regimens are summarized in table 1. Liver cirrhosis (Ishak staging 6) was not present in any patient. In cases with unavailable liver biopsy, cirrhosis was excluded by ultrasound examination.

**Table 1:** Baseline characteristics of the study cohort.

Parameter	
Gender (n)	
-Male	20 (61%)
-Female	13 (39%)
Age (years)	
-Mean ± SD	44±14
HBeAg status (n)	
-HBeAg positive	12 (36%)
-HBeAg negative	15 (45%)
Inactive HBsAg carrier (n)	6 (18%)
Ethnicity	
-Caucasian	27 (82%)
-Asian	4 (12%)
-Afro-American	2 (6%)
Genotype (n)	
-Genotype A	18 (54%)
-Genotype B	1 (3%)
-Genotype C	3 (9%)
-Genotype D	10 (30%)
-Genotype E	1 (3%)
Hepatic Fibrosis (Ishak staging) (n)	
F0	3 (9%)
F1	6 (18%)
F2	6 (18%)
F3	2 (6%)
F4	1 (3%)
F5	2 (6%)
F6	0
Not available	13 (39%)
Organ transplantation (n)	
-Liver transplantation	5 (15%)
-Renal transplantation	4 (12%)
Duration of NUC therapy (months)	
-Mean ± SD	34±21
-Median (range)	28 (11–111)
Treatment regimen (n)	
-Lamivudine (LAM) monotherapy	8
-Adefovir (ADV) monotherapy	6
-Telbivudine (LDT) monotherapy	1
-Entecavir (ETV) monotherapy	2
-Tenofovir (TDF) monotherapy	2
-Lamivudine+Adefovir	6
-Lamivudine/Entricitabine+Tenofovir	6
-Tenofovir+Entecavir	5

**Figure 1:**
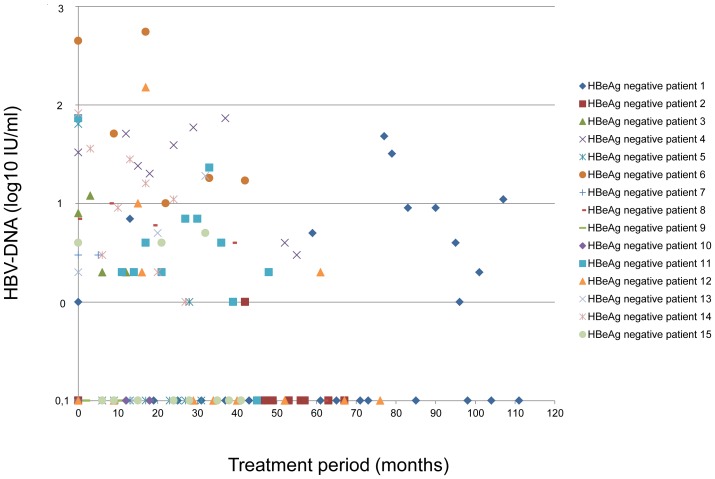
Minimal residual viremia in HBeAg negative individuals treated with nucleos(t)ide analogues.

The median number of tested serum samples per patient was six (range 2–31) during a period of 28 (range 11–111) months. Minimal residual viremia was detected in 31/33 individuals (n = 160/237 serum samples, 68%) and was significantly more frequently present in untreated HBsAg carriers (n = 20/21 serum samples, 95%) and NUC treated HBeAg positive (n = 67/78, 87%) compared to NUC treated HBeAg negative patients (n = 73/138, 53%) (p = 0.0002 and p = 0.0001, respectively) **(“** **Fig. 1** **,** **2** **, and** **3** **.”)**.

**Figure 2:**
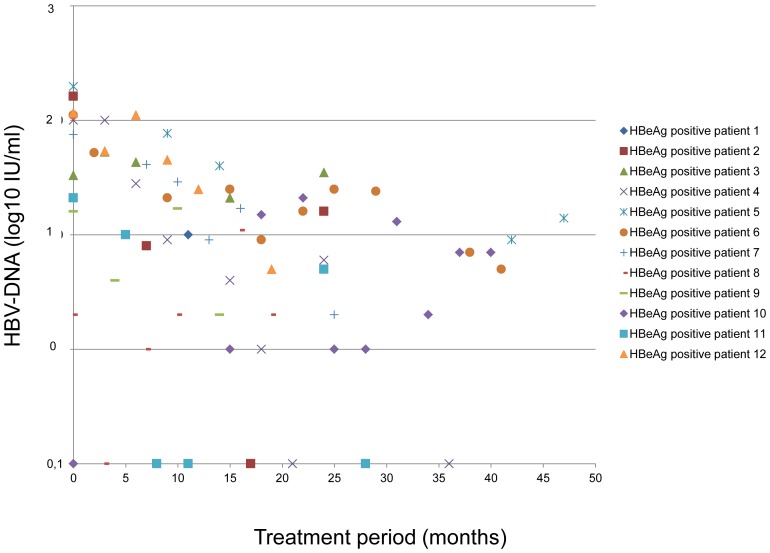
Minimal residual viremia in HBeAg positive individuals treated with nucleos(t)ide analogues.

**Figure 3:**
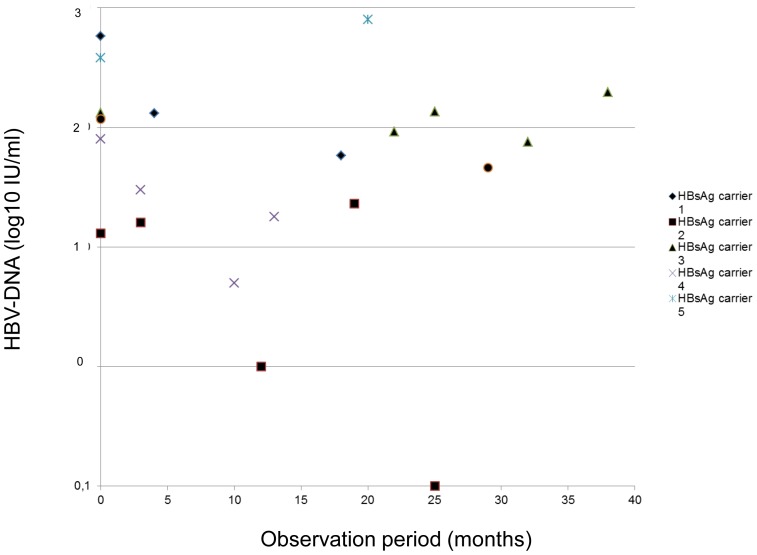
Minimal residual viremia in untreated HBsAg carriers.

Median MRV levels in HBsAg carriers, HBeAg positive and HBeAg negative patients were 78 (1–800), 16 (1–197) and 9 (1–549) IU/mL.

A complete suppression of HBV DNA as evaluated by real-time PCR was obtained in at least one sample in 20/33 patients. In contrast MRV was continuously detectable in five HBsAg carriers (83%), five HBeAg positive cases (42%), and four HBeAg negative individuals (25%) at all tested time points during a period of up to 38, 55, and 47 months, respectively.

### Minimal Residual Viremia According to Treatment Regimens

Minimal residual viremia according to treatment regimens is listed in table 2.

**Table 2:** Minimal residual viremia according to treatment regimen.

Treatment regimen	Median treatment duration(months)	Analyzed serumsamples(n)	Serum samples with minimalresidual viremia (%)
Lamivudine monotherapy	23 (5–84)	54	50%
Adefovir monotherapy	42 (11–76)	41	54%
Telbivudine monotherapy	19	6	83%
Entecavir monotherapy	33.5 (27–40)	21	95%
Tenofovir monotherapy	14 (9–19)	10	70%
Lamivudine+Adefovir	22 (3–37)	25	56%
Lamivudine/Emtricitabine+Tenofovir	27.5 (9–41)	42	81%
Tenofovir+Entecavir	15 (3–28)	17	65%

In serum samples of HBeAg positive patients, minimal residual viremia was not significantly more often observed during monotherapy with adefovir, telbivudine, entecavir, or tenofovir (n = 26/28, 93%) compared to combination therapy (lamivudine+adefovir, tenofovir+lamivudine/emtricitabine, or tenofovir+entecavir, n = 41/50, 82%) (p = 0.31). If only treatment regimens with the highly potent nucleos(t)ide analogues entecavir and tenofovir were considered a statistical difference between monotherapies (n = 17/18, 94%) and combination therapies (n = 39/48, 81%) could not be detected, either (p = 0.26).

Likewise to HBeAg positive patients, minimal residual viremia was not significantly more often observed during monotherapy with lamivudine, adefovir, entecavir, or tenofovir (n = 55/104, 53%) compared to combination therapy (lamivudine+adefovir, tenofovir+lamivudine/emtricitabine, n = 18/34, 53%) (p = 1.0) in serum samples of HBeAg negative individuals. If only treatment regimens with entecavir and tenofovir were considered a statistical difference between monotherapies (n = 10/13, 77%) and combination therapies (n = 6/11, 55%) could not be detected, either (p = 0.39).

### Minimal Residual Viremia in Patients with Organ Transplantation

Nine patients received antiviral therapy as part of the immunoprophylaxis after solid organ transplantation. Type and level of immunosuppression did not influence the prevalence of minimal residual viremia compared to immunocompetent patients (number of samples with MRV: n = 45/78 (58%) vs. n = 95/138 (69%), p = 0.1).

Minimal residual viremia was not significantly more often observed during monotherapy with lamivudine, entecavir, or tenofovir (n = 32/50, 64%) compared to combination therapy (lamivudine+adefovir, tenofovir+lamivudine, or tenofovir+entecavir, n = 13/28, 46%) (p = 0.16).

If only treatment regimens with entecavir and tenofovir were considered combination therapies (tenofovir+lamivudine, tenofovir+entecavir) suppressed HBV-DNA levels below the real-time PCR limit of detection more frequently (serum samples with MRV n = 13/24, 54%) than entecavir or tenofovir alone (serum samples with MRV n = 26/27, 96%; p = 0.0006).

### Clinical Relevance of Minimal Residual Viremia

Two patients during adefovir monotherapy and one individual during lamivudine therapy developed viral resistance (table 3). These three individuals were not under immunosuppressive therapy and displayed minimal residual viremia in all analysed serum samples prior to the occurrence of resistance against nucleos(t)ide analogues.

**Table 3:** Clinical characteristics of patients with viral resistance.

Patient	Ethnicity	HBeAg status	HBV-genotype	Treatment regimen	Duration of minimal residual viremia until viral resistance (months)	Serum samples with minimal residual viremia (n)	Viral mutation	HBV-DNA (IU/mL) at time of resistance
Patient 1	Caucasian	Negative	D	ADV	45	7/7	A181V,	81200
						(100%)	N236X	
Patient 2	Caucasian	Positive	A	ADV	21	4/4	N236T	71600
						(100%)		
Patient 3	Caucasian	Negative	A	LAM	31	5/5	M204I	4240
						(100%)		

**Figure 4:**
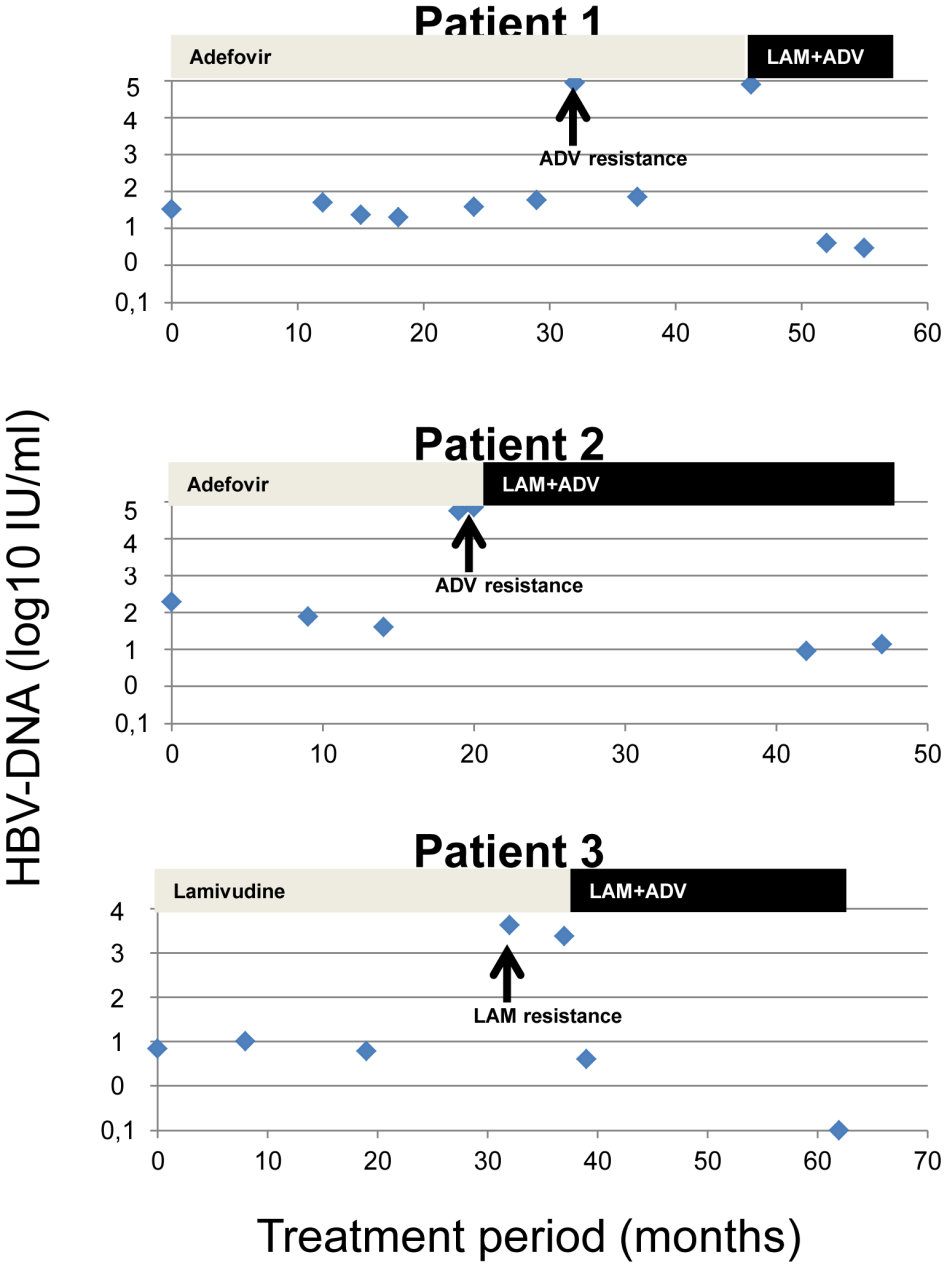
Minimal residual viremia and viral breakthrough in cases with adefovir and lamivudine resistance. Patients 1 and 2: Adefovir resistance. Patient 3: Lamivudine resistance.

At the time point of resistance HBV-DNA levels raised from 6×10^0^–1.97×10^2^ IU/mL to a maximum of 4.24×10^3^, 7.16×10^4^, and 8.12×10^4^ IU/mL, respectively. After initiation of combination therapy with lamivudine plus adefovir, HBV-DNA levels declined promptly, however, minimal residual viremia persisted in two of the three individuals **(“** **Fig. 4** **.”)**.

In one of the patients with adefovir resistance (table 3, patient 1), resistance analysis could be repeated in a post-hoc analysis in serum samples with MRV. 17 months prior to the time point when the adefovir related mutations A181V and N236X had been initially detected in clinical routine, post-hoc resistance analysis revealed wild type virus in a sample with 59 IU/mL HBV-DNA. However, in the following serum sample nine months later, the A181V mutation could be detected, although minimal residual viremia did not have significantly increased (73 IU/mL).

Except the three patients mentioned above, none of the other NUC-treated patients showed a confirmed>1 log HBV-DNA increase at two sequential time points to HBV-DNA levels>100 IU/mL.

Clinically, there was no evidence for disease deterioration with ALT elevations>2x the baseline values of individual patients. In cases with MRV and viral resistance, ALT levels were heterogenous: Of the patients with adefovir resistance, one did not show an ALT elevation, while the other experienced only a marginal ALT increase (0.86 µkat/L, normal<0.85 µkat/L). The case with lamivudine resistance had ALT levels 1.2x the upper limit of normal at the time of treatment failure.

## Discussion

In chronic hepatitis B, highly sensitive real-time PCR techniques with a lower limit of detection of 10–20 IU/mL [17]–[20] allow quantification of minimal residual viremia which could not be detected in the registration trials of the potent nucleos(t)ide analogues entecavir, telbivudine, and tenofovir which were performed with conventional PCRs with a lower limit of detection between 60–80 IU/mL [3]–[6]. Applying real-time PCR in clinical routine, clinicians become confronted with positive HBV-DNA results in patients who seemed to be successfully treated with NUCs so far and were always HBV-DNA negative determined by conventional PCR. Since current European treatment guidelines define long-term treatment response by the lower HBV-DNA threshold of 10–20 IU/mL and recommend treatment adjustment in case of ongoing viral replication (partial virologic response) in order to avoid viral resistance and treatment failure [2], the question arises whether successfully applied long-term treatment regimens should be adapted in daily clinical practice.

In our real-life cohort minimal residual hepatitis B viremia was present very frequently in 94% of patients and 68% of individual serum samples. It was significantly more present in HBeAg positive compared to HBeAg negative individuals and occurred during all different nucleos(t)ide analogue treatment regimens irrespective of mono- or combination therapy or the patients immune status.

The observation that prevalence of minimal residual viremia is not significantly different between mono- and combination therapies in immunocompetent individuals does not support the idea that a combination therapy including the highly potent nucleos(t)ide analogues entecavir and tenofovir is more effective than treatment with either drug alone. These results are in line with recently published controlled clinical trials, in which the combination entecavir plus tenofovir was not more effective than an entecavir monotherapy in treatment naïve patients or in which the combination tenofovir plus emtricitabine failed to be superior to a tenofovir monotherapy in treatment experienced individuals [15], [16]. Entecavir treated individuals who show HBV-DNA levels<1000 IU/mL after one year of therapy display a continuous decline of viral load during prolonged antiviral therapy and do not develop viral resistance [14]. Similar data are available from the long-term treatment arms of the tenofovir registration trials: Even in patients with high baseline viral load>10^9^ copies/mL HBV-DNA suppression below 400 copies/mL is only a matter of time without the risk of treatment failure if patients are adherent to therapy [9], [21]. Whether these observations can be extrapolated to individuals with immunosuppression after organ transplantation remains to be elusive so far. In our study, in contrast to immunocompetent patients, minimal residual viremia was significantly less frequently detected during combination therapies including entecavir or tenofovir compared to both drugs alone in patients receiving immunosuppressive therapy. Since patients under immunosuppression may be at higher risk for disease progression and HBV reactivation leading to liver cirrhosis and hepatocellular carcinoma, the relevance of minimal residual viremia should be further evaluated in this special patient population.

Treatment failure caused by viral resistance did not occur in any individual treated with entecavir, telbivudine, or tenofovir, but in three cases receiving lamivudine or adefovir monotherapy. Both drugs are known to select viral mutations during long-term therapy much more frequently than entecavir or tenofovir [22]. Interestingly, in the one patient in whom resistance analysis could be performed in our post-hoc approach adefovir resistance could not be detected in a serum sample with 59 IU/mL HBV-DNA, but at the subsequent time point, at which HBV-DNA had only marginally increased to 73 IU/mL. Thus, even very low viral replication may cause viral resistance during treatment with antivirals with low barrier to resistance, and a “safe limit” of viral replication should not be defined.

We therefore feel that detection of minimal residual viremia during prolonged monotherapy with low barrier to resistance drugs like lamivudine or adefovir should provoke treatment adjustment in order to avoid viral resistance. In contrast, monotherapy with highly potent nucleos(t)ide analogues like entecavir and tenofovir does not need treatment adaptation in case of minimal viremia.

It should be noted that our study cohort did not include patients with liver cirrhosis. Thus, the clinical relevance of minimal residual viremia should not be extrapolated to this patient group. Complete suppression of viral replication remains a major goal of antiviral therapy to prevent decompensation of cirrhosis and lower the risk of hepatocellular carcinoma [2], [23].

In summary, minimal residual viremia is frequent during long-term NUC-therapy of chronic hepatitis B. Viral resistance occurs during lamivudine or adefovir monotherapy, but not with entecavir or tenofovir. In immunocompetent patients, a combination therapy based on entecavir or tenofovir is not associated with a lower prevalence of minimal residual viremia compared to monotherapy regimens. Additional studies should prove whether HBV-DNA suppression below the real-time PCR threshold is mandatory for highly potent NUCs.
